# Combining virtual model and cone beam computed tomography to assess periodontal changes after anterior tooth movement

**DOI:** 10.1186/s12903-018-0635-y

**Published:** 2018-11-01

**Authors:** Sun-Hyun Kim, Jong-Bin Lee, Min-Ji Kim, Eun-Kyoung Pang

**Affiliations:** 1 Department of Clinical Oral Health Science, Graduate School of Clinical Dentistry, Ewha Womans University, Seoul, Korea; 2 Department of Periodontology, Mokdong Hospital, Ewha Womans University, Seoul, Korea; 3 Department of Orthodontics, School of Medicine, Ewha Womans University, Seoul, Korea; 4 Department of Periodontology, School of Medicine, Ewha Womans University, 1071 Anyangcheon-ro, Yangcheon-gu, Seoul, 07985 Republic of Korea

**Keywords:** CBCT, Virtual model, Gingival thickness, Alveolar bone thickness

## Abstract

**Background:**

Orthodontic force may affect not only periodontal ligaments, but also the alveoloar bone and the gingiva according to the type of tooth movements. The authors assessed changes in gingival thickness (GT) and alveolar bone thickness (ABT) after orthodontic treatment using a new method.

**Methods:**

This study included 408 teeth (208 central incisors, 200 lateral incisors) from the upper and lower 4 anterior teeth of 52 patients who had completed orthodontic treatment. GT and ABT were measured using virtual casts fabricated from impressions and cone beam computed tomography (CBCT). Two sectioned images of every tooth axis were acquired by partitioning each tooth with a line connecting the midpoint of the incisal edge to the midpoint of the cementoenamel junction in the virtual models and the root apex in CBCT images. After superimposing the two sectioned images, GT and ABT were measured before and after orthodontic tooth movement. Correlations between GT and ABT before and after treatment, and changes in GT and ABT associated with sex, tooth arch, tooth position, orthognathic surgery, and tooth inclination and rotation were assessed.

**Results:**

Before orthodontic treatment, GT and ABT were significantly correlated. Patients who underwent orthognathic surgery exhibited an increase in GT thickness compared with those who did not. ABT was significantly decreased in proclined teeth and in rotated teeth.

**Conclusions:**

GT and ABT can be affected by the nature of tooth movement and can be accurately assessed by comparing sectioned CBCT images and virtual models.

## Background

Recent economic growth has prompted an increase in the demand for orthodontic treatment, particularly, interest in pursuing improvement of the aesthetic aspects of physiognomy. In the past, the purpose of orthodontic treatment was simple tooth alignment; however, aesthetic improvement in physiognomy, completion of occlusion, and the aesthetics of periodontal tissue have also become important factors in responding to patient demands. Therefore, it is necessary to diagnose and prevent undesirable changes in surrounding tissues associated with orthodontic treatment [1–3].

Although orthodontic treatment offers many benefits to patients including tooth alignment, establishment of occlusion, and aesthetic improvement of physiognomy, large amounts of tooth movement can cause unexpected adverse side effects such as root resorption, bone dehiscence, bone perforation, and gingival recession [4, 5]. The loss of alveolar bone is also a potential side effect of orthodontic treatment. When a tooth and its surrounding tissue are moved by orthodontic force(s) and the root compresses the periodontium for a certain period of time, the alveolar bone can be resorbed at that point, and bone is deposited where tension is created [6]. Alveolar bone loss can differ in various ways, including magnitude, direction, and duration of orthodontic force(s). Orthodontic force(s) affects not only periodontal ligaments and roots, but also alveolar bone. If it is beyond the physiological range of force suitable for a tooth of a tooth and its surrounding tissues, side effects, such as root resorption and alveolar bone resorption, may occur after orthodontic treatment [7].

It has been reported that in patients with a high alveolar crest and thin alveolar bone, there is a high possibility of recession and loss of alveolar bone at the anterior labial and lingual aspects of the cortical bone [8]. When the anterior teeth move, bone reconstruction occurs in the alveolar crest or bone surrounding the root apex. However, bone reconstruction rarely occurs at the apical region near the cortical plate or mandibular symphysis [9, 10]. From the biological perspective of orthodontic treatment, the thickness of alveolar bone anchoring a tooth, as well as the amount of tooth movement, are important factors when devising an orthodontic plan and choosing an orthodontic treatment method [11]. The thickness of alveolar bone can has been associated with skeletal growth patterns and could be altered by grafting procedures [12, 13].

Many previous studies have measured gingival thickness (GT) and alveolar bone thickness (ABT) [14]. Generally, methods can be divided into those that are invasive and involve tissue damage, such as bone sounding, and those that are non-invasive, such as cone beam computed tomography (CBCT), although radiation exposure may be an issue [15]. One invasive method involves locally anesthetizing the patient’s mouth, then measuring thickness directly by inserting a periodontal probe, a needle, or an endodontic instrument [16]. This method has disadvantages in that the patient must endure the pain associated with the anesthesia and measurement; moreover, the results of this method are also prone to inter-observer/operator variation [17]. Conversely, the non-invasive method using CBCT is relatively accurate; however, it may be subject to error when soft tissues, such as the gingiva, are measured due to limits of its resolution [18, 19]. Therefore, we introduce a new method, which combined CBCT imaging to measure ABT and the images generated using a digital intraoral scanner to measure GT. Using measurement data thus acquired, we studied correlations between GT and ABT before and after orthodontic treatment, and the changes in GT and ABT associated with various factors including sex, tooth arch, tooth position, orthognathic surgery, and tooth inclination and rotation.

## Methods

This retrospective study involved a total of 408 teeth (208 central and 200 lateral incisors). Specifically, a total of 416 teeth were evaluated from 4 upper and lower anterior teeth of 52 patients who had available records of orthodontic treatment for malocclusion at the Ewha Womans University Mokdong Hospital between January 2009 and April 2016. A total of 408 teeth were selected according to the inclusion criterion that GT and ABT could be measured in natural teeth without defects. Teeth in which GT and ABT could not be measured because of defects, such as those treated with previous orthodontic treatment or periodontal surgery, were excluded. A total of 8 teeth were excluded: 5 that were extracted for treatment; 2 that were replaced by implants; and 1 exhibiting a congenital defect.

This study was approved by the Ethics Committee of the School of Medicine, Ewha Womans University Medical Center (Approval number: EUMC 16–05–032-005).

### Measurement of GT and ABT

#### Cast scanning

**Fig. 1:**
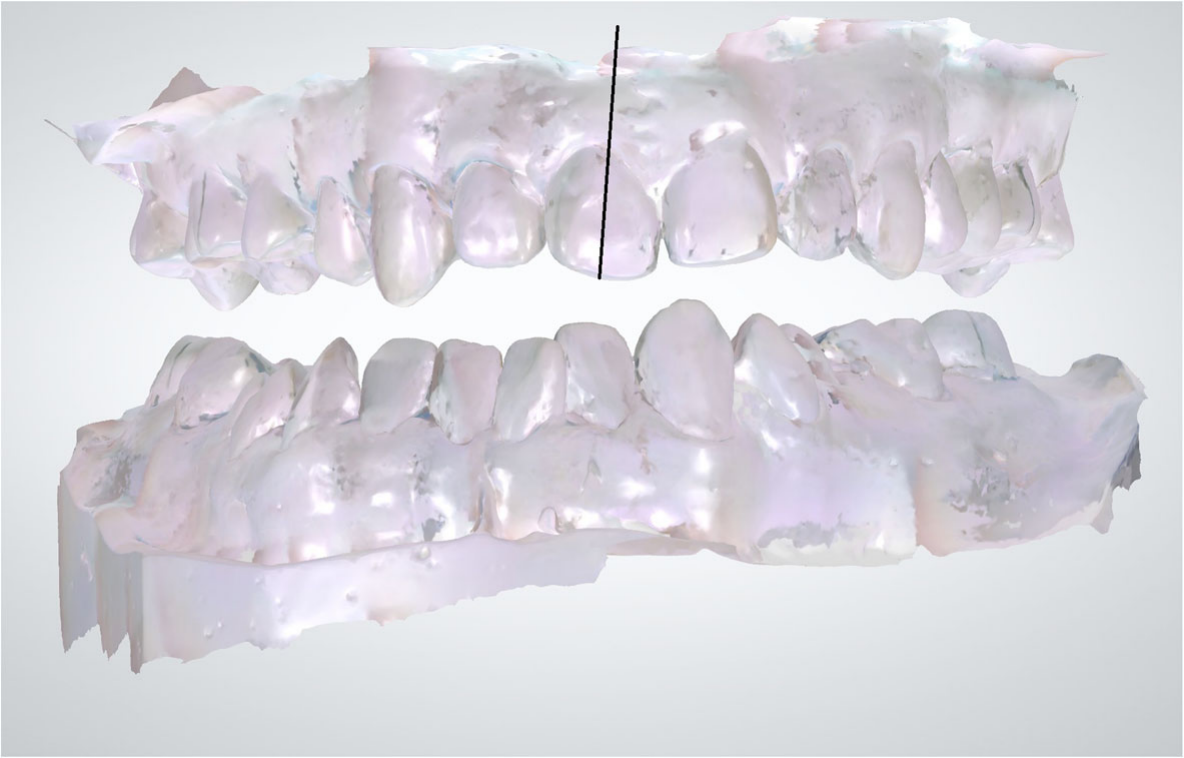
Tooth sectioning in a cast. After scanning a cast with a digital intraoral scanner, the anterior teeth were sectioned with the line connecting the midpoint of the incisal edge and the midpoint of the cemento enamel junction

After scanning each patient’s cast with a digital intraoral scanner (Trios Pod, 3shape dental systems, Copenhagen, Denmark), each anterior tooth in the virtual model was sectioned via a line connecting the midpoint of the incisal edge and the midpoint of the cementoenamel junction (CEJ) (Fig. 1). Tooth section images were acquired for 4 upper and lower anterior teeth. This type of cast scanning is different from intraoral scanning in that patient cast impressions were scanned using an intraoral scanner.

#### CBCT

CBCT was performed using a Dinnova3 device (HDXWILL, Chung-ju, Korea) with the following operating parameters: current, 10 mA; voltage, 85 kVp; minimum voxel size, 0.15 mm.

**Fig. 2:**
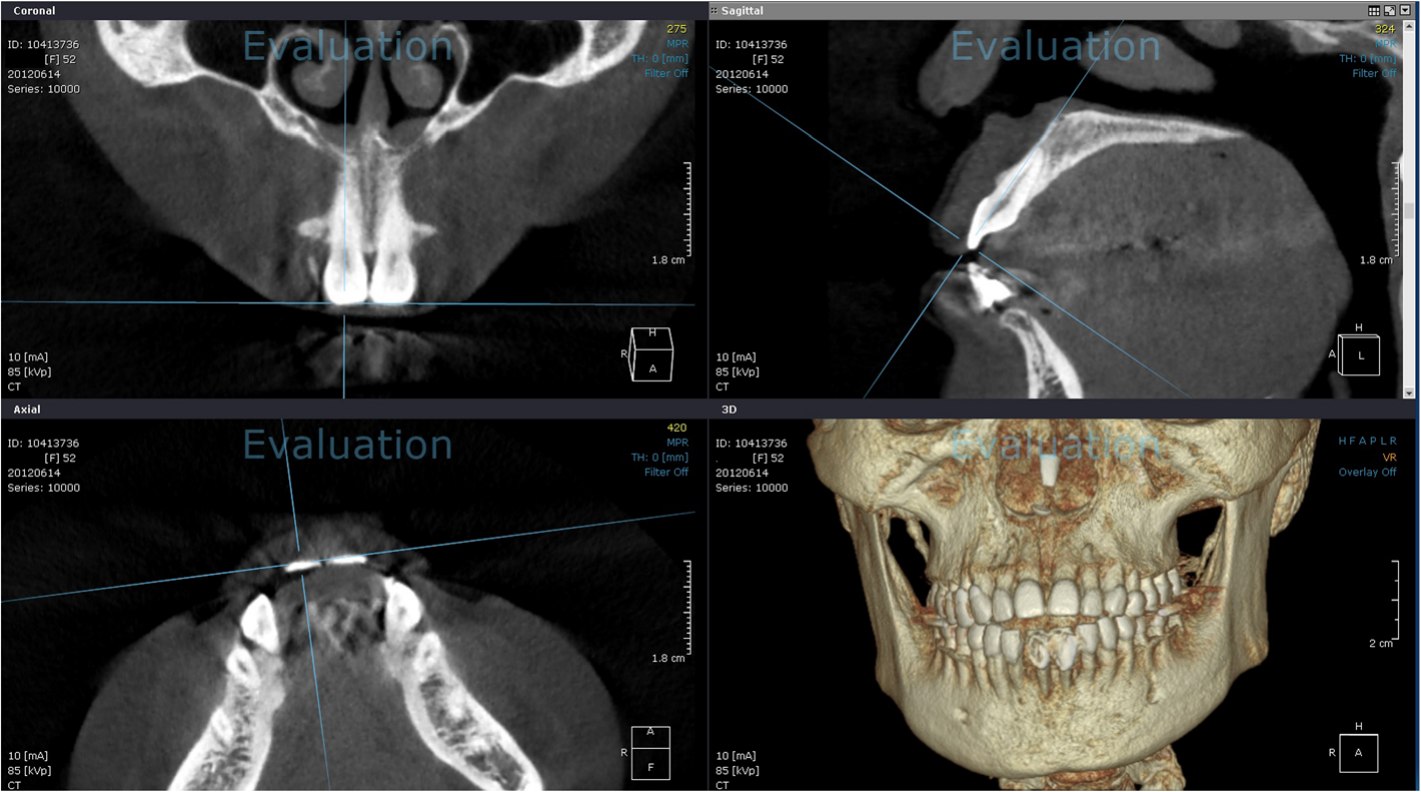
Tooth sectioning via cone beam computed tomography. The image was obtained by sectioning each tooth along the line connecting the midpoint of the incisal edge and the root apex, using software (OnDemand 3D)

Each CBCT image was acquired by sectioning via a line connecting the midpoint of the incisal edge and the root apex using software (OnDemand 3D, Cybermed Co., Seoul, Korea) for each of 4 upper and lower anterior teeth (Fig. 2).

#### Superimposition

**Fig. 3:**
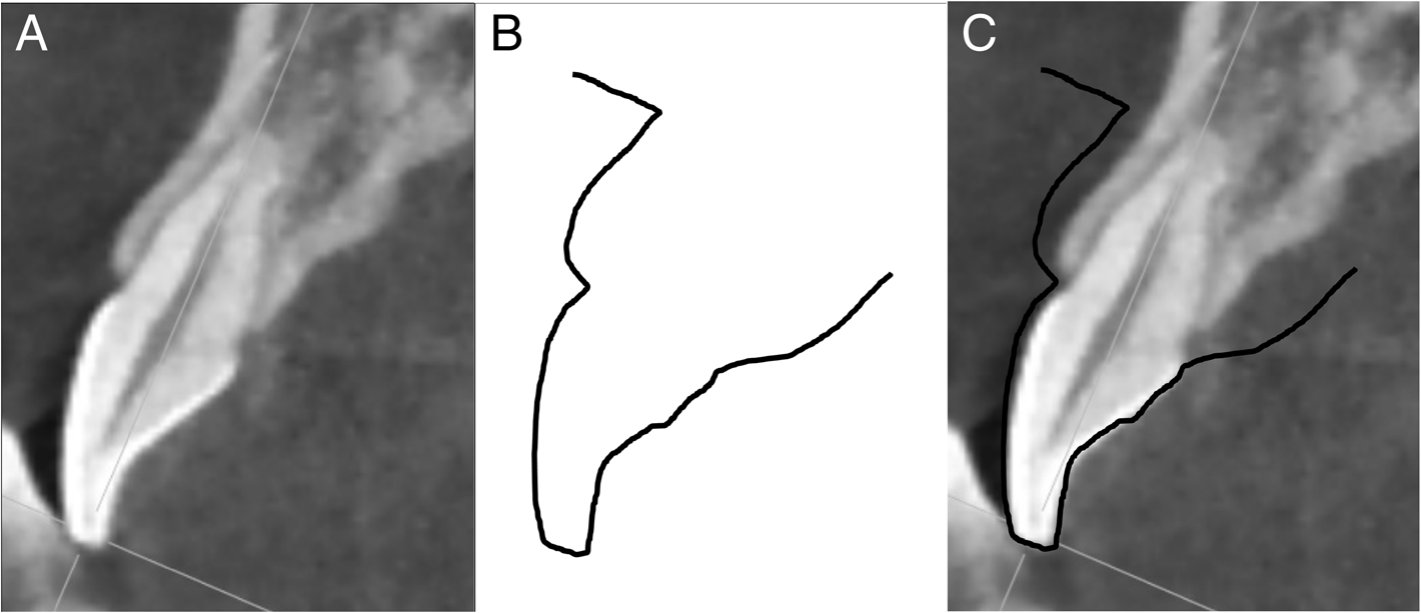
Superimposition of cast and cone beam computed tomography images. **a** The cone beam computed tomography image. **b** The scanned cast image. **c** Superimposition of (**a**) and (**b**)

**Fig. 4:**
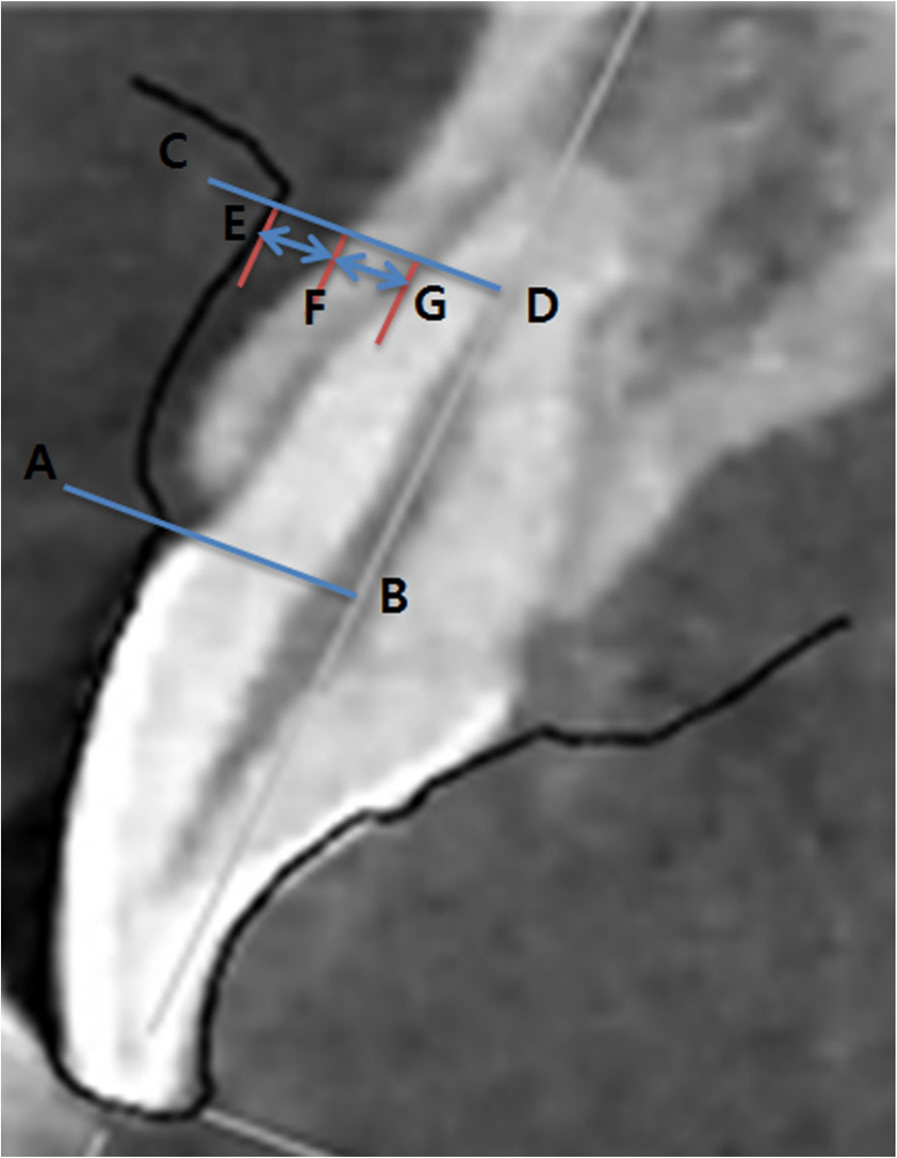
Measurement of gingival thickness and alveolar bone thickness. **a**, **b**: Perpendicular line from the cemento enamel junction to the tooth axis. **c**, **d** The line incorporating parallel translation of (**a**, **b**) towards its root apex as 4 mm. **e**, **f** Gingival thickness. **f**, **g** Alveolar bone thickness

Sectioned CBCT images and those from the virtual models were superimposed to match the shape of the clinical crown of each tooth using software (Photoshop CS, Adobe, San Jose, CA, USA) (Fig. 3). To measure GT and ABT, a perpendicular line was drawn to the tooth axis from a point that was 4 mm apical to the CEJ (Fig. 4). This superimposition method was similar to a technique used in a previous study [14] and has been validated. Furthermore, to ensure reliability, all measurements were performed by the same trained operator, and repeated 3 times per case for superimposition and measurement of GT and ABT. Sufficiently reliable data were obtained (*p* <  0.05).

### Measurement of tooth inclination and rotation

#### Inclination

**Fig. 5:**
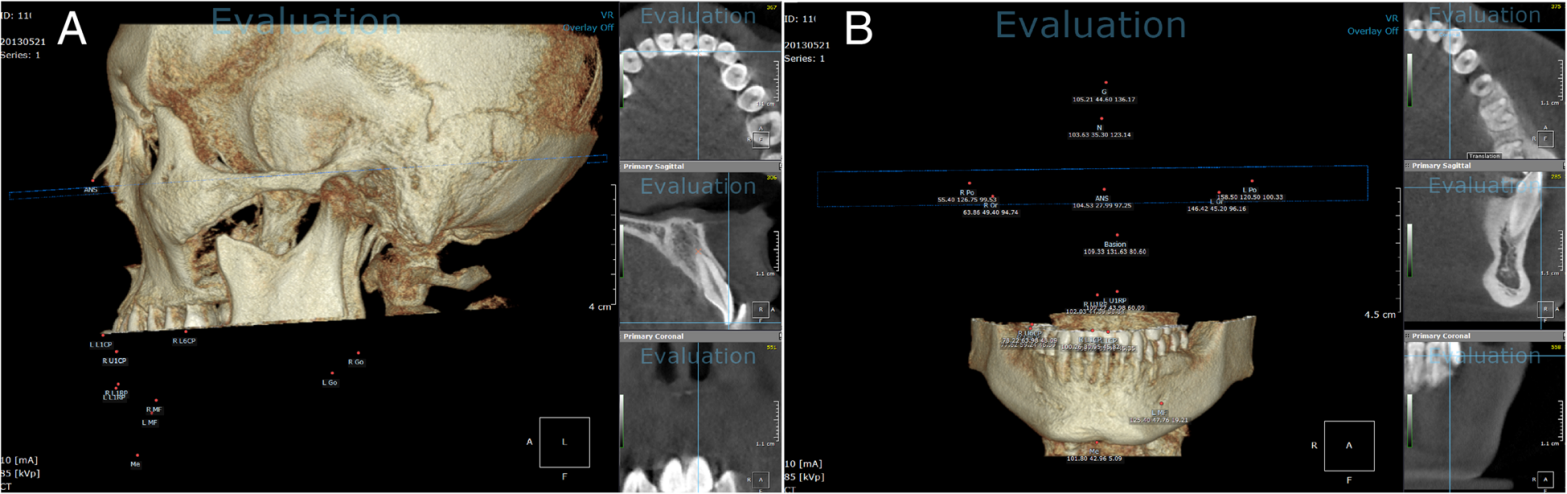
Measurement of tooth inclination with (**a**) the angle between the Frankfurt horizontal plane and the axis of the tooth in the maxilla, and (**b**) the angle between the mandibular plane and the axis of the tooth in the mandible

To measure tooth inclination, patients were divided into two groups: those who underwent orthognathic surgery and those who did not. In those who underwent orthognathic surgery, the axes of the maxillary teeth were measured in the Frankfort horizontal plane and those of the mandibular teeth were measured in the mandibular plane. In those who never underwent orthognathic surgery, the axes of both the maxillary and the mandibular teeth were measured in the occlusal plane (Fig. 5).

#### Rotation

**Fig. 6:**
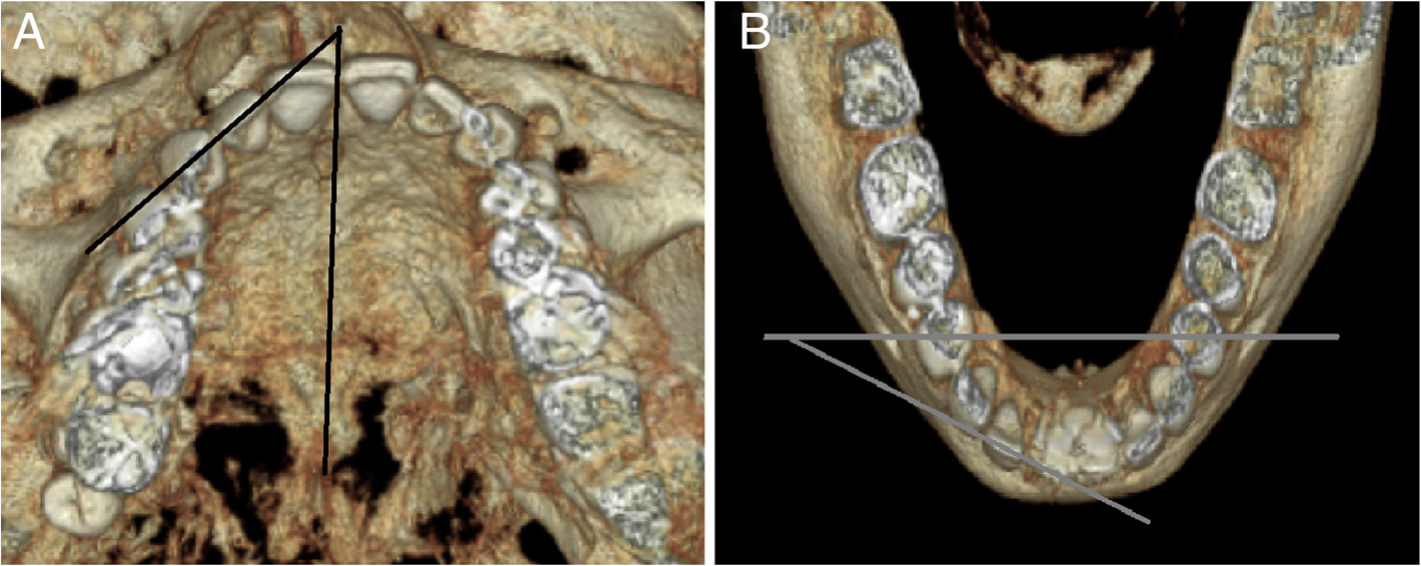
Measurement of tooth rotation with (**a**) the angle between the median palatine suture and the extended line of the incisal edge of the tooth in the maxilla, and (**b**) the angle between the line connecting the mental foramen and the extended line of the incisal edge of the tooth in the mandible

To assess the amount of tooth rotation, the angle between the median palatine suture and the extended line of the incisal edge in the maxilla, and the angle between the line connecting both right and left mental foramen and the extended line of the incisal edge in the mandible, was measured. Clockwise tooth rotation was defined as positive (+), while counterclockwise tooth rotation was defined as negative (−). The measured angles before and after orthodontic treatment were analyzed (Fig. 6).

### Analysis of GT and ABT

After measuring GT and ABT by superimposing the sectioned image of the virtual model over the CBCT sectioned image, the results were analyzed as described below.

#### Correlations between GT and ABT

The correlations between GT and ABT before and after orthodontic treatment were analyzed and compared.

#### Changes in GT and ABT before and after orthodontic treatment

The changes in GT and ABT associated with sex, tooth arch (maxilla/mandible), tooth position (central/lateral), orthognathic surgery (with/without), and tooth inclination (proclination/retroclination) before and after orthodontic treatment were analyzed and compared.

#### Changes in GT and ABT associated with tooth movement

After dividing tooth movement according to inclination or rotation, changes in GT and ABT associated with tooth inclination or rotation were analyzed and compared.

### Statistical analysis

The data showed normal distribution. Pearson’s correlation analysis was performed to analyze correlations between GT and ABT before and after orthodontic treatment. The independent *t*-test was then used to analyze changes in GT and ABT associated with sex, tooth arch, tooth position, orthognathic surgery, and tooth inclination before and after orthodontic treatment. Finally, multi-linear regression analysis was performed to investigate changes in GT and ABT associated with tooth inclination and tooth rotation. All statistical analyses were performed using SPSS version 20 (IBM Corporation, Armonk, NY, USA); *p* <  0.05 was considered to be statistically significant in all tests.

## Results

A total of 408 teeth, from 4 upper and lower anterior teeth of 52 patients, were analyzed. The mean age of was 22 years, with the following distribution: teenagers (49.5%); twenties (35.0%); thirties (9.8%); and forties and older (5.6%). The mean duration of treatment was 2 years 1 month, and the sex distribution was 80.4% female and 19.6% male (Table 1).

**Table 1:** Patient characteristics

Variable	N	%
Age:	mean 21.78 ± 8.77 years	
10–19	202	49.50
20–29	143	35.04
30–39	40	9.80
40 and over	23	5.63
Treatment period	mean 2.19 ± 0.16, years	
Gender		
Male	80	19.60
Female	328	80.39
Tooth arch		
Maxilla	203	49.75
Mandible	205	50.25
Tooth position		
Central incisor	208	50.98
Lateral incisor	200	49.02
Orthognathic surgery of the tooth		
With Surgery	205	50.24
Without surgery	203	49.75

*N* number of teeth

There was a significant correlation between GT and ABT, which was evident only before orthodontic treatment (*p* <  0.05) (Table 2). Changes in GT were not significantly associated with sex, tooth arch (maxilla/mandible), tooth position (central/lateral), or tooth inclination (i.e., proclination/retroclination) before and after orthodontic treatment. However, the change in GT was statistically significant in patients who underwent orthognathic surgery (*p* <  0.05) (Table 3). Changes in ABT were not significantly associated with sex, tooth arch, tooth position, orthognathic surgery, or tooth inclination before and after orthodontic treatment. However, ABT had significantly decreased in teeth that were proclined (*p* < 0.05) (Table 4).

**Table 2:** Correlations between gingival thickness and alveolar bone thickness before and after orthodontic treatment

	r	*p*-value
Before	0.194	< 0.001
After	0.055	0.268

*r* Pearson’s correlation coefficient

**Table 3:** Changes in gingival thickness before and after orthodontic treatment

Variable	Δ	*p* value
Gender		
Male	0.047	0.316
Female	0.103	
Tooth arch		
Maxilla	0.025	0.140
Mandible	0.091	
Tooth position		
Central incisor	0.062	0.867
Lateral incisor	0.054	
Orthognathic surgery of the tooth		
With Surgery	0.143	< 0.001
Without surgery	−0.027	
Translation		
Proclination	0.048	0.597
Retroclination	0.072	

Δ = amount of gingival thickness change after orthodontic treatment

**Table 4:** Change in alveolar bone thickness before and after orthodontic treatment

Variable	Δ	*p* value
Gender		
Male	−0.013	0.268
Female	0.031	
Tooth arch		
Maxilla	0.002	0.200
Mandible	0.043	
Tooth position		
Central incisor	0.031	0.599
Lateral incisor	0.142	
Orthognathic surgery of the tooth		
With Surgery	0.003	0.223
Without surgery	0.042	
Translation		
Proclination	−0.089	0.021^*^
Retroclination	0.065	

Δ = amount of alveolar bone thickness changes after orthodontic treatment

^*^p < 0.05

Changes in GT and ABT associated with tooth inclination and rotation were analyzed and compared. In the multilinear regression analysis, GT was not statistically associated with tooth inclination or rotation (Table 5). Similarly, ABT was also not statistically associated with tooth inclination (neither proclination nor retroclination); however, it was significantly associated with tooth rotation (*p* < 0.05). Specifically, greater tooth rotation was associated with a greater reduction in ABT (Table 6).

**Table 5:** Changes in gingival thickness by tooth movement

Variable	Standardized coefficient, β	95% CL		*p* value
Translation	0.004	0.000	0.008	0.064
Rotation	0.000	−0.003	0.004	0.808

*CL* confidence limits

**Table 6:** Changes in alveolar bone thickness by tooth movement

Variable	Standardized coefficient, β	95% CL		*p* value
Translation	0.001	−0.004	0.007	0.588
Rotation	−0.004	−0.006	− 0.001	0.005^*^

*CL* confidence limits

^*^p < 0.05

## Discussion

Interest in non-invasive methods to assess and/or measure soft tissue has increased because of patient pain and resistance to orthodontic diagnosis or periodontal treatment [20]. We aimed to measure GT and ABT using non-invasive methods that do not damage tissue. Although many studies investigating the anatomical shape of the gingiva, the depth of periodontal pockets, and the length of the junctional epithelium have been published [17, 21], relatively few studies examining GT have been reported. It has been suggested that this is partly due to the difficulty of measuring GT [22]. To improve the measurement of GT, we developed a new method using a sectioned image of the patient’s virtual dental model. Images acquired using sectioned virtual dental models and volume-rendered CBCT were superimposed, and subtractive analysis was subsequently used to measure GT and ABT. According to previous studies, the alveolar bone crest is usually located 4 mm below the CEJ. Therefore, we drew a perpendicular line to the tooth axis from a point that was 4 mm apical to the CEJ [23], and measured GT and ABT on that line.

Opinions differ as to whether orthodontic treatment causes gingival recession [24–27]. Periodontal disease caused by poor oral conditions during orthodontic treatment may occasionally cause gingival recession; however, loss of alveolar bone due to tooth movement also causes gingival recession. Many previous studies have reported major reasons for gingival recession caused by tooth movement after orthodontic treatment, which included a free gingival margin < 0.5 mm, thin alveolar bone or dehiscence, proclination, and orthognathic surgery [24–27]. Given the results of the present study, we additionally conclude that proclination causes reduced ABT. However, the results relating to changes in GT and ABT before and after orthodontic treatment and their association with sex and orthognathic surgery (with/without) in the current study, differed from those of previous studies. Notably, only 19.2% (80 of 408) of the teeth in the current study were from male subjects, which is one potential reason for the discrepancy in results [15, 28]. With regard to orthognathic surgery, we suggest that the discrepancy may be due to the different measurement method used [29, 30]. In the current study, the angles between the tooth axis and each plane were measured. In patients who underwent orthognathic surgery, the inclination of maxillary teeth was measured in the Frankfort horizontal plane and that of the mandibular teeth was measured in the mandibular plane. In patients who never underwent orthognathic surgery, the inclination of both maxillary and mandibular teeth was measured in the occlusal plane. In contrast, previous studies have used a variety of measurement methods such as ANB (A point, nasion, B point), the angle between the SN line and FH line, among others. We believe that these differences in measurement methods influenced the results [31]. We did not consider the exact type of orthognathic surgery because several previous studies have reported that orthognathic surgery has no effect on the gingiva [32–34].

The current study had some limitations, the first of which was its retrospective design and the use of casts from patients who had already completed orthodontic treatment. If direct intraoral scanning—rather than casting—could have been performed, the results of the study would have been more accurate and reliable. In general, alginate impressions are made at the beginning and at the end of orthodontic treatment. Although possible deformation of casts made from alginate impressions has been reported, it has advantages including convenience and clinically acceptable accuracy [35]. Furthermore, many previous studies have reported high accuracy and reproducibility of digital models obtained by indirectly scanning dental casts [36, 37]. Notably, we suggest that the potential error arising from deformation did not significantly affect the results of the current study. In the future, we believe that further studies can be conducted using scanned images obtained directly from patient mouths via a digital intraoral scanner.

## Conclusions

In the present study, GT was decreased in patients who underwent previous orthognathic surgery, and ABT was decreased in cases of proclination. GT and ABT can be accurately assessed by comparing sectioned CBCT images and virtual models.

## Abbreviations

ABT: Alveolar bone thickness
ANB: A point, nasion, B point
CBCT: Cone beam computed tomography
CEJ: Cemento enamel junction
FH line: Frankfort horizontal line
GT: Gingival thickness
SN line: Sella nasion line
